# Distinct nucleotide patterns among three subgenomes of bread wheat and their potential origins during domestication after allopolyploidization

**DOI:** 10.1186/s12915-020-00917-x

**Published:** 2020-12-02

**Authors:** Yan Zhao, Luhao Dong, Conghui Jiang, Xueqiang Wang, Jianyin Xie, Muhammad Abdul Rehman Rashid, Yanhe Liu, Mengyao Li, Zhimu Bu, Hongwei Wang, Xin Ma, Silong Sun, Xiaoqian Wang, Cunyao Bo, Tingting Zhou, Lingrang Kong

**Affiliations:** 1 State Key Laboratory of Crop Biology, Shandong Key Laboratory of Crop Biology, College of Agronomy, Shandong Agricultural University, Tai’an, 271018 Shandong People’s Republic of China; 2 Key Laboratory of Crop Heterosis and Utilization, Ministry of Education, Beijing Key Laboratory of Crop Genetic Improvement, China Agricultural University, Beijing, 100193 People’s Republic of China; 3 Department of Bioinformatics and Biotechnology, Government College University, Faisalabad, 38000 Pakistan

**Keywords:** Bread wheat, Allopolyploidization, Evolution, Base composition, Subgenome divergence, DNA repair

## Abstract

**Background:**

The speciation and fast global domestication of bread wheat have made a great impact on three subgenomes of bread wheat. DNA base composition is an essential genome feature, which follows the individual-strand base equality rule and [AT]-increase pattern at the genome, chromosome, and polymorphic site levels among thousands of species. Systematic analyses on base compositions of bread wheat and its wild progenitors could facilitate further understanding of the evolutionary pattern of genome/subgenome-wide base composition of allopolyploid species and its potential causes.

**Results:**

Genome/subgenome-wide base-composition patterns were investigated by using the data of polymorphic site in 93 accessions from worldwide populations of bread wheat, its diploid and tetraploid progenitors, and their corresponding reference genome sequences. Individual-strand base equality rule and [AT]-increase pattern remain in recently formed hexaploid species bread wheat at the genome, subgenome, chromosome, and polymorphic site levels. However, D subgenome showed the fastest [AT]-increase across polymorphic site from *Aegilops tauschii* to bread wheat than that on A and B subgenomes from wild emmer to bread wheat. The fastest [AT]-increase could be detected almost all chromosome windows on D subgenome, suggesting different mechanisms between D and other two subgenomes. Interestingly, the [AT]-increase is mainly contributed by intergenic regions at non-selective sweeps, especially the fastest [AT]-increase of D subgenome. Further transition frequency and sequence context analysis indicated that three subgenomes shared same mutation type, but D subgenome owns the highest mutation rate on high-frequency mutation type. The highest mutation rate on D subgenome was further confirmed by using a bread-wheat-private SNP set. The exploration of loci/genes related to the [AT] value of D subgenome suggests the fastest [AT]-increase of D subgenome could be involved in DNA repair systems distributed on three subgenomes of bread wheat.

**Conclusions:**

The highest mutation rate is detected on D subgenome of bread wheat during domestication after allopolyploidization, leading to the fastest [AT]-increase pattern of D subgenome. The phenomenon may come from the joint action of multiple repair systems inherited from its wild progenitors.

**Supplementary information:**

The online version contains supplementary material available at 10.1186/s12915-020-00917-x.

## Background

The evolution of bread wheat (*Triticum aestivum*, AABBDD) is a complex process, due to that it is involved in a special hybrid speciation and subsequent global domestication and improvement [1–3]. Recent studies indicate that bread wheat originated from hybridization between cultivated tetraploid emmer wheat (*Triticum turgidum.* L, AABB) and wild diploid *Aegilops tauschii* (DD) around Fertile Crescent, and was further domesticated and improved in the process of spreading to the whole world [1, 2, 4, 5]. During the domestication after allopolyploidization, the three subgenomes of bread wheat went through profound changes, including generation of new mutations, insertions and deletions of fragment, genome-wide recombination, and massive alien introgressions [6, 7]. DNA base composition is an essential genomic feature that impacts codon usage, speciation, genome organization, and phylogenetic inference [8–10]. Recent studies documented the base-composition difference and mutation rate difference between populations separated by either domestication or demographic bottleneck event, which provide novel insights into genome evolution [11, 12]. Thus, the investigation of DNA base composition on bread wheat and its wild progenitors may be a better way to gain insights into divergent patterns of the genome/subgenomes during bread wheat domestication.

DNA base composition in organisms always follows some fixed rules. The Chargaff first parity rule (PR1) (i.e., [A] = [T] and [G] = [C]) is a common rule in a DNA duplex [13]. For each individual strand of a DNA duplex, second parity rule (PR2) (i.e., [A] ≈ [T] and [G] ≈ [C]) is detected and further verified by a large-scale study using 2210 species with sequenced whole genomes [11, 14, 15]. Furthermore, base composition follows the PR2 rule not only on the genome and chromosome levels but also on the polymorphic site level [11, 12]. Recently, a conserved base-composition pattern, [AT]-increase (i.e., modern accessions having significantly higher [A] and [T] values across genome-wide polymorphic sites than accessions sampled from their wild relatives), is discovered with natural populations across multiple species [11, 12]. Further study on regional variation of genome change pattern indicates that non-genic part of the genome has a greater contribution than genic SNPs to the [AT]-increase observed between wild and domesticated accessions in maize and soybean, and the separation between wild and domesticated accessions in [AT] values is significantly enlarged in non-genic and pericentromeric regions [12]. For the A, B, and D subgenomes of bread wheat, their ancestral genomes diverged several million years ago, followed by aggregation into the whole genome of bread wheat [16]. Therefore, it would be interesting to study and compare the subgenome change patterns of bread wheat during domestication after allopolyploidization.

Mutation type and mutation rate are two key factors impacting genome variation, which vary in different species, populations, and environments [17–19]. The human genome study shows that the DNA replication fidelity has not remained stable even since the origin of modern humans and might have changed numerous times during our recent evolutionary history [17]. One of the important rules for mutation is the mutation bias, i.e., mutations have a bias in the direction of A or T [20, 21]. Further analysis of data from multiple mutation accumulation experiments, either accumulating spontaneous or induced mutations, demonstrated higher [AT] values across mutation sites in derived lines at the end of mutation experiments than in ancestral lines, which suggested that base-composition difference can emerge from mutation sites [11]. Another important finding on mutation is that CpG dinucleotides are mutational hotspots, which are driven by frequent deamination of methylated cytosines [22, 23]. Transition frequency and sequence context analyses show the change from 5′-PyCG-3′ to 5′-PyTG-3′ is high frequency in maize and soybean, where Py is either a pyrimidine C or T [12]. So far, the mutation type and mutation rate of A, B, and D subgenomes of bread wheat remain unknown. A detailed understanding of the mutation spectrum on the three subgenomes is instrumental to studies of the mechanism of bread wheat genome change during domestication after allopolyploidization.

The DNA repair system is important to maintenance of balance between individual genome integrity and population genetic variability [24, 25]. Hypermutated genome leads to developmental disorders, deformities, and even death [26]. Additionally, some appropriate sequence changes provide population genetic variability to adapt biotic and abiotic stress under natural conditions [25, 27]. Recently, hundreds of DNA repair related genes have been identified in humans, which suggest a complex and integrated DNA repair system [28, 29]. Plants appear to have evolved a set of distinct checkpoint regulators in response to different types of stress on DNA especially solar-UV radiation, although over the last two decades it has become evident that the basic cell cycle toolbox of plants shares several similarities with those of fungi and mammals [30]. Recently, drawing this huge DNA repair system in detail is still hard, but it can be sure that there might be a DNA repair system within each donor of bread wheat genome, including emmer wheat and *Ae. tauschii*. However, the impact of the multiple DNA repair systems on bread wheat genome is still confounded. Thus, systematic analyses on DNA base composition, mutation rate, and DNA repair system of bread wheat are conducive to understand how the three subgenomes of bread wheat co-regulate individual genome integrity and population genetic variability.

In this study, we reported the genome/subgenome change pattern from wild progenitors to bread wheat, captured by base composition of bread wheat (AABBDD), durum (AABB), wild emmer (AABB), and *Ae. tauschii* (DD) summarized from data of sequence and polymorphic sites. One interesting finding was the fastest [AT]-increase on D subgenome from *Ae. tauschii* to bread wheat than these on A and B subgenomes from wild emmer to bread wheat. To reveal the cause of distinct subgenome change from wild progenitors to bread wheat, we analyzed and compared the [AT] values among three subgenomes within bread wheat and its wild progenitors from multiple perspectives, including different chromosomal windows, different functional annotation sets, and selective sweeps and non-selective sweeps. However, our results suggested that the fastest [AT]-increase of D subgenome was not caused by particular chromosomal regions or functional annotation sets, or selection. Furthermore, we compared mutation type and mutation rate among three subgenomes from wild progenitors to bread wheat. Three subgenomes shared the same mutation types, but D subgenome showed the highest mutation rates on the high-frequency mutation types. Using base composition of D subgenome as the phenotype, genome-wide scan showed the key loci for [AT]-increase of D subgenome were not only on D subgenome, suggesting an adjustment function of genome-wide cooperation. Together, these findings show systematically nucleotide change patterns within each of the three subgenomes from wild progenitors to bread wheat, as well as important loci accounting for nucleotide change patterns.

## Results

### D subgenome presents the largest variation of base composition during bread wheat domestication after allopolyploidization

**Fig. 1:**
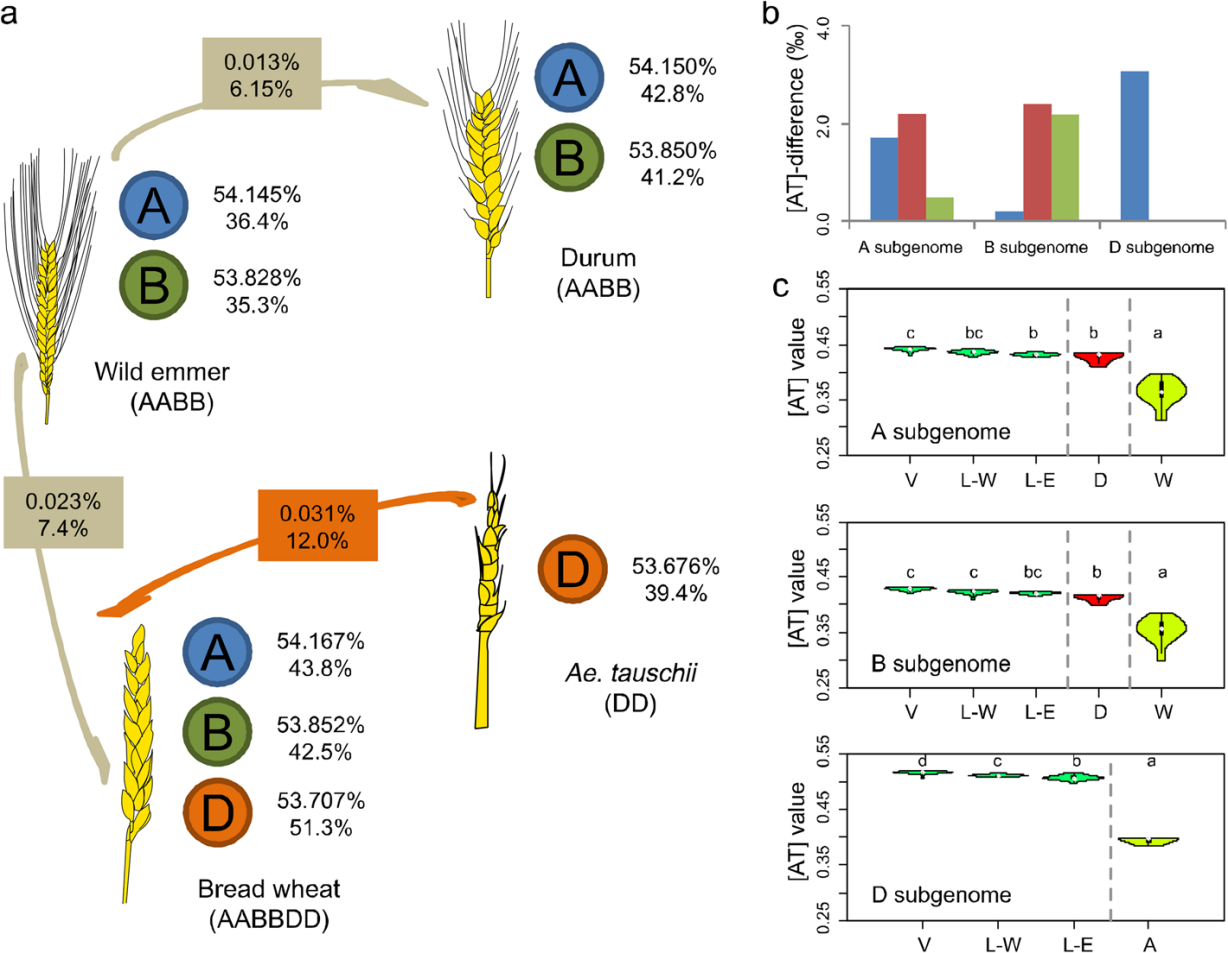
Distinct [AT]-increase patterns of bread wheat at genome and polymorphic site levels. **a** [AT]-increase pattern model of bread wheat during domestication after allopolyploidization. For each subgenome of each species, adjacent percentages show [AT] values at levels of genome (upper) and polymorphic site (bottom). The percentages with background color indicate average [AT]-increase from wild to domesticated accession. **b** Comparison of [AT] values of each subgenome among bread wheat (Chinese Spring), durum (Svevo), wild emmer (Zavitan), and *Ae. tauschii* (AL8/78). Blue bars show the differences of [AT] values between bread wheat and wild emmer in A and B subgenomes, and between bread wheat and *Ae. tauschii* in D subgenome; red bars show the differences of [AT] values between durum and wild emmer; and green bars show the differences of [AT] values between bread wheat and durum. **c** The [AT] values across polymorphic sites of bread wheat and its wild progenitors (including bread wheat variety (V), landrace-west (L-W), landrace-east (L-E), durum (D), wild emmer (W), and *Ae. tauschii* (A)). Different letters above the violins indicate significant differences (*p* < 0.05) when analyzed by Duncan’s test

The genome sequences of bread wheat (AABBDD, Chinese Spring), durum (AABB, Svevo), wild emmer (AABB, Zavitan), and *Ae. tauschii* (DD, AL8/78) were obtained to compare the genome/subgenome-wide base compositions between bread wheat and its diploid and tetraploid progenitors (see the “Methods” section) [31–34]. For each species above, single-strand parity rule 2 (PR2), i.e., [A] ≈ [T] and [C] ≈ [G], is applicable to base compositions of genome, subgenome, and even the single chromosome (Additional file 1: Fig. S1). Meanwhile, we obtained an [AT] value for each of three subgenomes within each accession (see the “Methods” section). For each of three subgenomes, the significantly higher [AT] values were detected in bread wheat (A, 54.167%; B, 53.852%; and D, 53.707%) and durum (A, 54.150%, and B, 53.850%) when compared with wild emmer (A, 54.145%, and B, 53.828%) and *Ae. tauschii* (D, 53.676%) by a simulation test (Fig. 1a and Additional file 1: Figs. S2 and S3) (see the “Methods” section). The findings agreed with that the [AT]-increase, i.e., [AT] value in wild progenitors, is less than that in domesticated accessions for each subgenome. In general, we confirmed that the PR2 and [AT]-increase remained stable in recently formed hexaploid species bread wheat at genome-wide. However, D subgenome showed higher growth of [AT] value (0.031%, from *Ae. tauschii* to bread wheat) than those in A and B subgenomes (0.022% and 0.024%, from wild emmer to bread wheat), suggesting about 1.35× growth of [AT] value on D subgenome than that on A and B subgenomes (Fig. 1a, b). It would be interesting to explore the changes of [AT] value across polymorphic site among the three subgenomes of the bread wheat population during domestication after allopolyploidization.

### D subgenome presents the fastest [AT]-increase across polymorphic sites during bread wheat domestication after allopolyploidization

To explore base-composition changes across polymorphic site from wild progenitors to bread wheat, we analyzed the published resequencing data of 93 accessions worldwide, including 20 from wild emmer, 5 from *Ae. tauschii*, 5 from durum, 15 from bread wheat landrace-east, 14 from bread wheat landrace-west, and 34 from bread wheat varieties [6]. There are a total of 34,802,951 and 33,300,925 SNPs in A and B subgenomes of bread wheat, wild emmer, and durum, respectively, together with 16,491,115 SNPs in D subgenome of bread wheat and *Ae. tauschii*. Upon eliminating loci with low minor allele frequency (MAF) or high missing rate (see the “Methods” section), SNPs were selected for analyses as a common subset within bread wheat and its wild progenitors. The PR2 across polymorphic site was further identified in each accession of the mixed population at subgenomic/chromosomal polymorphic site level (Additional file 1: Figs. S4 and S5). For A and B subgenomes, the [AT] values across polymorphic site were highest among the three bread wheat groups, varieties (44.2% and 42.8%), landrace-west (43.6% and 42.3%), and landrace-east (43.2% and 42.1%), followed by durum (42.8% and 41.2%), whereas wild emmer had the lowest [AT] values of A and B subgenomes at 36.4% and 35.3% (Fig. 1a, c, and Additional file 2: Table S1). For D subgenome, the [AT] values were also higher in the three bread wheat groups, varieties (51.7%), landrace-west (51.1%), and landrace-east (50.7%), whereas *Ae. tauschii* had lower [AT] value at 39.4% (Fig. 1a, c, and Additional file 2: Table S1). The results showed that the individual-strand base compositions across polymorphic sites on each subgenome all follow [AT]-increase from wild progenitors to bread wheat (including landraces and improved varieties).

Through horizontal comparisons of [AT] values across polymorphic site among three subgenomes, it is interesting to find that the [AT] value of D subgenome (51.3%) is significantly higher than those of A and B subgenomes (43.8% and 42.5%) in bread wheat, with an average difference of 8.2% (Fig. 1a, c). Meanwhile, we also detected that [AT] value of *Ae. tauschii* (39.4%, DD) was significantly higher than that of wild emmer (35.9%, AABB) (Fig. 1a, c, and Additional file 2: Table S1). Further vertical comparison indicated that the [AT]-difference on D subgenome (12.0%) between bread wheat and *Ae. tauschii* is the largest than those on A (7.4%) and B (7.2%) subgenomes between bread wheat and wild emmer. The results suggested that there is the fastest [AT]-increase across polymorphic site on D subgenome from *Ae. tauschii* to bread wheat than those on A and B subgenomes from wild emmer to bread wheat.

### The fastest [AT]-increase across polymorphic sites on the D subgenome is not dependent on any particular chromosome segments

**Fig. 2:**
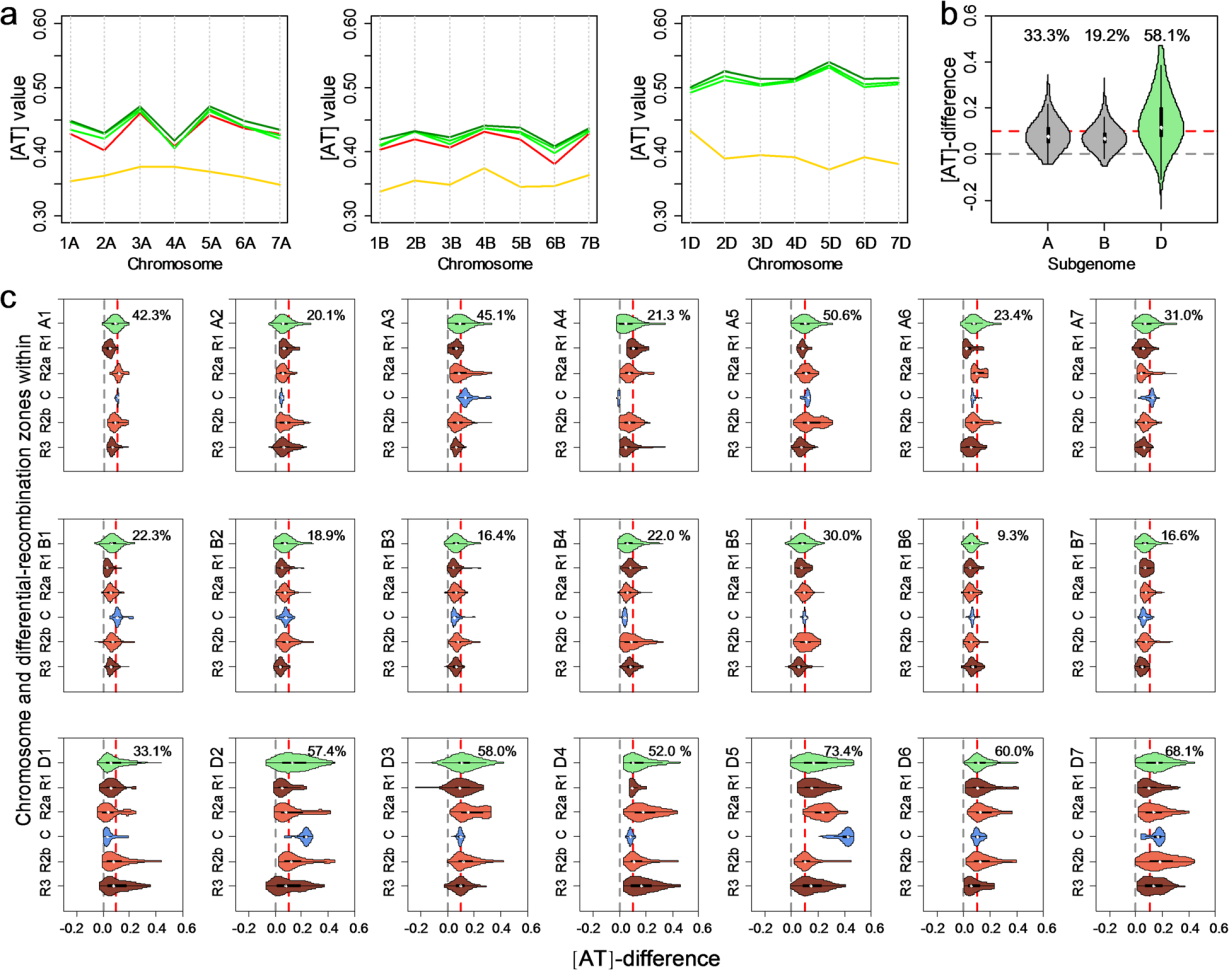
Distinct [AT]-increase among subgenomes from wild progenitors to bread wheat at chromosome and chromosomal window levels. **a** Chromosomal [AT] values of bread wheat and its wild progenitors. Three groups of bread wheat including variety, landrace-west, and landrace-east are colored dark green, green, and light green, respectively. Wild emmer and *Ae. tauschii* are colored yellow, while durum is colored red. **b** [AT]-differences across 2-Mb windows between bread wheat and wild emmer on A and B subgenomes (gray), and between bread wheat and *Ae. tauschii* on D subgenome (green). Gray horizontal line at [AT]-difference = 0 indicates the separation of chromosomal windows following [AT]-increase pattern (98.6%, 13,693/13,879) and others. Red horizontal line is another important boundary at [AT]-difference = 0.1, due to that there are less chromosomal windows above the line in A and B subgenomes (26.1%, 2599/9962), but more in D subgenome (58.1%, 2277/3917). Numbers above violins are the proportions of chromosomal window above the red horizontal line. **c** [AT]-difference between bread wheat and its wild progenitors on 21 chromosomes and 105 differential-recombination regions. *X*-axis indicates [AT]-difference, and the *Y*-axis indicates chromosome and differential-recombination regions within. For chromosomal violins, the adjacent numbers are the proportion of chromosomal window above the red horizontal line. For differential-recombination zones, the adjacent letters indicate significant differences (*p* < 0.05) when analyzed by Duncan’s test

In order to determine whether the fastest [AT]-increase across polymorphic sites of D genome is caused by the base-composition change in specific genome regions, we scan the base-composition distribution along whole genome. Firstly, we calculated and compared [AT] values of each chromosome in three subgenomes of 93 accessions mentioned above. Chromosomal [AT] values of bread wheat were higher than the corresponding chromosomal [AT] values of wild emmer in A and B subgenomes, 9.5 to 26.9% and 8.5 to 24.0%, respectively (Fig. 2a). Additionally, chromosomal [AT] values of durum were also higher than those of wild emmer in A and B subgenomes, 8.5 to 24.0% and 10.1 to 19.4%, respectively (Fig. 2a). For D subgenome from *Ae. tauschii* to bread wheat, the chromosomal [AT] value had increased by 15.6 to 44.2% (Fig. 2a). Generally, D subgenome showed the fastest [AT]-increase across polymorphic sites than those in A and B subgenomes at chromosome level.

We further analyzed and compared the [AT] value across polymorphic sites for each 2-Mb chromosome windows (the sliding step was 1 Mb) between bread wheat and its wild progenitors (see the “Methods” section). Firstly, we noticed that [AT] values of bread wheat were consistently higher than those of its wild progenitors at each of almost all chromosome windows (98.6%, 13,693/13,879), which confirmed that [AT]-increase was a general rule along whole genome during bread wheat domestication after allopolyploidization (Fig. 2b and Additional file 1: Figs. S6 and S7). Secondly, [AT]-differences between bread wheat and its wild progenitors on D subgenome are significantly larger than those on A and B subgenomes at chromosomal window level (Fig. 2b and Additional file 1: Figs. S6 and S7). For most of chromosome windows on A and B subgenomes (73.9%, 7363/9962), the [AT]-differences between bread wheat and wild emmer ranged from 0 to 10% (Fig. 2b). By comparison, half of the chromosome windows on D subgenome (58.1%, 2277/3917) showed [AT]-differences between bread wheat and *Ae. tauschii* for more than 10% (Fig. 2b). The results suggested that the fastest [AT]-increase of D subgenome could be evolutionary pattern of base compositions within entire D subgenome, which is not determined by a few special chromosome windows.

Given that the recombination was a basic genome feature [31], we further examined the relationship between recombination pattern and [AT]-increase on chromosomal window level. All of the 105 main differential-recombination regions on 21 chromosomes showed [AT]-increase (3.73 to 17.63%) except for one proximal region (C) on chromosome 4A (− 0.38%) (Fig. 2c and Additional file 1: Fig. S7). Among 42 distal regions (R1 and R3), 22 (A, 8; B, 7; and D, 7) showed the lowest [AT]-increase on corresponding chromosomes with significance, whereas 8 (A, 3; B, 3; and D, 2) occupied the fastest [AT]-increase. Meanwhile, 9 (A, 2; B, 3; and D, 4) out of 21 proximal regions showed the significantly lowest [AT]-increase, while that of 7 regions (A, 2; B, 3; and D, 2) was opposite (Fig. 2c and Additional file 1: Fig. S7). Further comparison showed that there were no significant differences in the contribution of proximal and distal regions to [AT]-increase at the levels of genome and subgenome (Additional file 1: Fig. S8).

### [AT]-increase of three subgenomes is mainly contributed by intergenic regions at non-selective sweeps

**Fig. 3:**
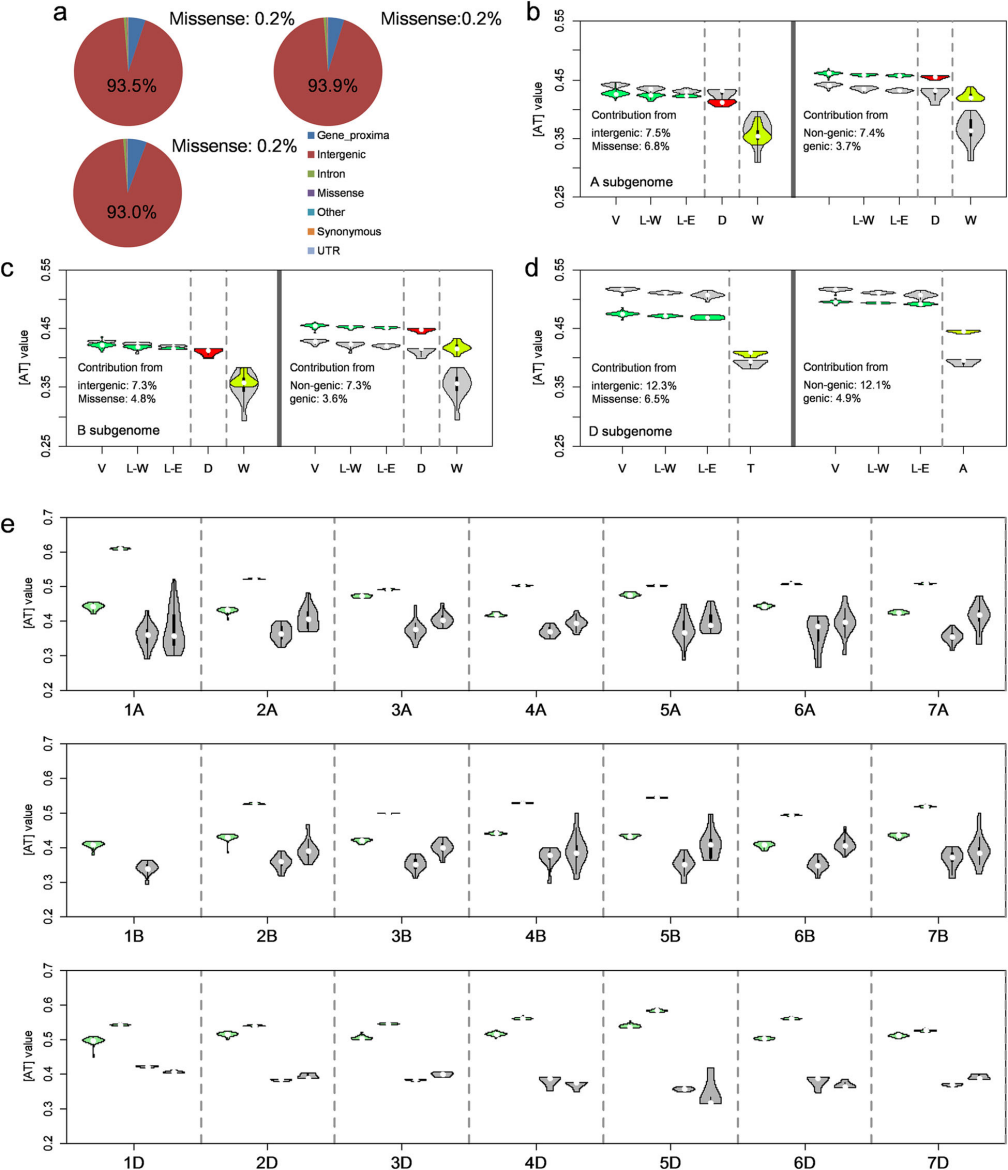
Base-composition pattern of different functional annotation sets. **a** The proportions of SNPs within 7 annotation sets. Comparison of [AT] values between intergenic SNPs and missense SNPs (left), and between non-genic SNPs and genic SNPs (right) in A (**b**), B (**c**), and D (**d**) subgenomes. For each plot, gray violins show [AT] values of intergenic SNPs (left) and non-genic SNPs (right), whereas the other colors indicate missense SNPs (left) and genic SNPs (right). The percentages within each plot show the contributions on [AT]-increase from wild progenitors to bread wheat. **e** Comparison of [AT] values between non-sweeps and sweeps on each chromosome. Both blue violins in each plot show [AT] values calculated with non-sweep SNPs (left) and sweep SNPs (right) of bread wheat landraces. Both gray violins in each plot show [AT] values calculated with non-sweep SNPs (left) and sweep SNPs (right) of wild emmer (A and B subgenomes) and *Ae. tauschii* (D subgenome)

Change of base composition between bread wheat and its wild progenitors could be affected by artificial selection on functional loci for agronomic traits [12]. Here, we classified the genome-wide SNPs into 7 functional annotation sets (intergenic, gene-proximal, UTRs, intronic, synonymous, missense, and other genic) [35] and compared the base composition of two representative sets among the three subgenomes in bread wheat (see the “Methods” section). The first one was intergenic SNP set representing slight effect on gene function, which appeared to be 93.5%, 93.9%, and 93.0% of A, B, and D subgenomes, respectively (Fig. 3a). The other was missense SNP set accounting for protein sequence, which had a small proportion in A (0.2%), B (0.2%), and D (0.2%) subgenomes (Fig. 3a). [AT]-increases from wild progenitors to bread wheat were consistently observed by using intergenic SNPs and missense SNPs within each of the three subgenomes (Fig. 3b–d). The [AT]-differences between bread wheat and its wild progenitors at intergenic SNPs (A, 7.5%; B, 7.3%; and D, 12.3%) were larger than that at missense SNPs (A, 6.8%; B, 4.8%; and D, 6.5%) within three subgenomes, especially D subgenome (Fig. 3b–d). The results were supported by a simulative test (Additional file 1: Fig. S9). To further explore the contributions from different functional annotation sets to the [AT]-increase, we combined intergenic and gene-proximal sets to form the non-genic SNP set and combined the rest five original genomic annotation sets to form the genic SNP set. As expected, [AT]-increases from wild progenitors to bread wheat remained by using non-genic SNP set and genic SNP set within each of the three subgenomes (Fig. 3b–d). Further simulation test also agreed with that non-genic SNP set (A, 7.4%; B, 7.3%; and D, 12.1%) have greater contributions to the [AT]-increase than that of genic SNP set (A, 3.7%; B, 3.6%; and D, 4.9%) within three subgenomes (Fig. 3b–d and Additional file 1: Fig. S9). These results suggested that the [AT]-increase of three subgenomes, especially the D subgenome, was determined by [AT]-increase of intergenic SNPs rather than these SNPs with significant influence on gene function.

To test the effect of selective sweeps on [AT]-increase, we investigated the rate of base composition among 546 reported domestication-related selective sweeps (54.6 Mb) on three subgenomes [6]. As expected, no significant [AT]-difference was detected between selective and non-selective sweeps within each chromosome of bread-wheat’s wild progenitors (Fig. 3e). For each chromosome of bread wheat, the [AT] values at selective sweeps were obviously larger than that at non-selective sweeps, suggesting direct impact on overall [AT]-increase from artificial selection (Fig. 3e). Further comparison of selective sweeps among three subgenomes indicated that there was significantly but slightly higher [AT] value on D (55.0%) than those on the other two subgenomes (A, 52.0%, and B, 51.8%), with an average difference of 3.9% (Fig. 3e and Additional file 1: Fig. S10). For non-selective sweeps, the [AT] value on D subgenome (51.1%) was also significantly higher than the other two subgenomes (A, 44.4%, and B, 42.5%), with an average difference of 7.65% (Fig. 3e and Additional file 1: Fig. S10). Larger [AT]-differences at non-selective sweeps of D subgenome between bread wheat and *Ae. tauschii* suggested that non-selective sweeps were major contributors for the fastest [AT]-increase of D subgenome during bread wheat domestication after allopolyploidization.

### [AT]-increase of three subgenomes is caused by same mutation types

The fastest [AT]-increase of D subgenome could be caused by its unique mutation signatures including mutation type and/or rate. So we examined the contribution from each of the 6 transition types of bi-allelic SNPs on [AT]-increase (see the “Methods” section). A/G and C/T were two major transition types and had similar frequencies in A subgenome (35.7% and 35.7%), B subgenome (35.0% and 35.0%), and D subgenome (33.4% and 33.4%). (Additional file 1: Fig. S11). Another two [AT]-increase related transition types, T/G and A/C, also occupied similar proportions in A subgenome (8.4% and 8.4%), B subgenome (8.8% and 8.8%), and D subgenome (9.1% and 9.2%) (Additional file 1: Fig. S11).

**Fig. 4:**
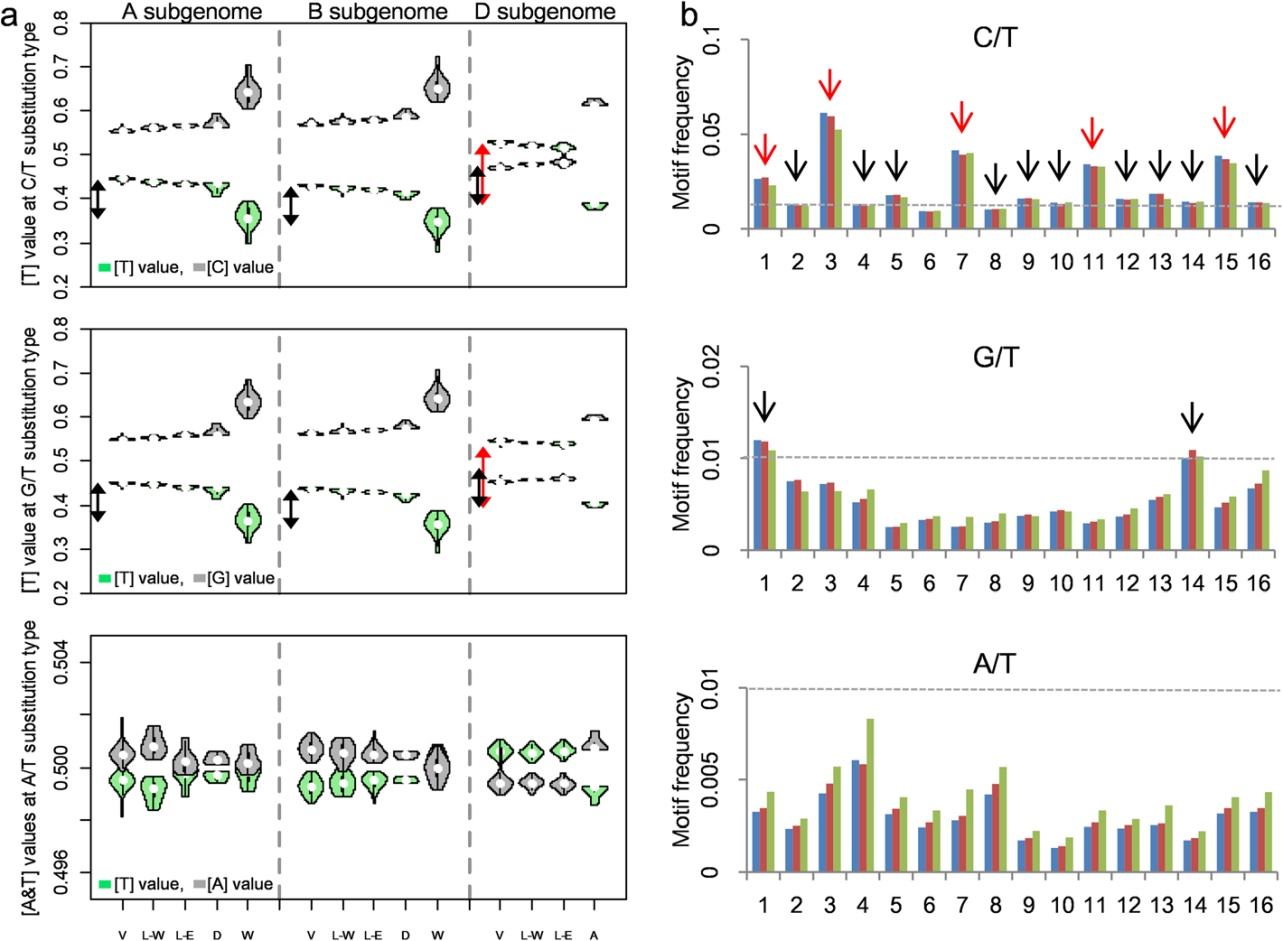
Base-composition distribution and frequency of tri-nucleotide motifs at each of three representing transition types. **a** The [T] values at each transition type of C/T (top), G/T (middle), and A/T transition type (bottom) within bread wheat and its wild progenitors (including bread wheat variety (V), landrace-west (L-W), landrace-east (L-E), durum (D), wild emmer (W), and *Ae. tauschii* (A)). For each transition type, the violins of three subgenomes are plotted together and separated by dashed lines. For each plot, the length of black arrow presents the [T]-difference on corresponding transition type of A or B subgenome between bread wheat and wild emmer, whereas the length of red arrow presents the [T]-difference on corresponding transition type of D subgenome between bread wheat and *Ae. tauschii*. **b** Frequency of 48 motifs at C/T (top), G/T (middle), and A/T transition type (bottom). For each plot, blue, red, and green bars show A, B, and D subgenome, respectively. The numbers 1–16 on *Y*-axis show 16 motifs, 5′-ANA-3′, 5′-ANC-3′, 5′-ANG-3′, 5′-ANT-3′, 5′-CNA-3′, 5′-CNC-3′, 5′-CNG-3′, 5′-CNT-3′, 5′-GNA-3′, 5′-GNC-3′, 5′-GNG-3′, 5′-GNT-3′, 5′-TNA-3′, 5′-TNC-3′, 5′-TNG-3′, and 5′-TNT-3′ in order, where N is the corresponding transition type. The horizontal line in each plot indicated the expected frequency at 0.01 (≈ 1/96). The 17 motifs with frequency above the threshold are marked by arrows, including 5 motifs with frequency more than twice the threshold marked by red arrows. Correspondingly, the reverse and complementary 48 motifs around A/G, A/C, and C/G types are also provided in Fig. S13

We then calculated and compared the contributions of each transition type to [AT]-increase among the three subgenomes. For A subgenome, bread wheat had more bases A or T at A/G, C/T, G/T, and A/C transition types than wild emmer at 8.4%, 8.4%, 8.3%, and 8.4%, respectively (Fig. 4a and Additional file 1: Fig. S12). Meanwhile, significant [A&T]-difference between bread wheat and wild emmer was also identified at A/G (8.3%), C/T (8.2%), G/T (8.0%), and A/C (8.0%) transition types on B subgenome, respectively (Fig. 4a and Additional file 1: Fig. S12). By contrast, there were the largest [A&T]-differences between bread wheat and *Ae. tauschii* at A/G (14.0%), C/T (14.1%), G/T (14.1%), and A/C (14.3%) transition types on D subgenome (Fig. 4a and Additional file 1: Fig. S12). In general, contributions of the 4 transition types above could explain the overall [AT]-increase within each subgenome. The highest proportional increases in A or T of 4 transition types in D subgenome could be the fundamental cause of higher [AT]-increase of D subgenome.

Given that SNPs occurred more frequently in certain sequence contexts, we classified SNPs and their adjacent upstream and downstream into 96 tri-nucleotide motifs, and compared the frequency of tri-nucleotide motifs among the three subgenomes. As with the 6 transition types mentioned above, no obvious differences were detected in proportions of the 96 motifs among three subgenomes, with *r* ≥ 0.95 between any two of three subgenomes (Fig. 4b and Additional file 1: Fig. S13). The results suggested that the three subgenomes shared the same mutation types. We further identified 34 high-frequency motifs, with each having a frequency for more than expected frequency at 0.01 (1/96) in each of the three subgenomes (Fig. 4b and Additional file 1: Fig. S13). Interestingly, 30 out of the 34 motifs were around C/T and A/G transition types, including 5′-ANA-3′, 5′-ANC-3′, 5′-ANG-3′, 5′-ANT-3′, 5′-CAN-3′, 5′-CNG-3′, 5′-CNT-3′, 5′-GNA-3′, 5′-GNC-3′, 5′-GNG-3′, 5′-GNT-3′, 5′-TNA-3′, 5′-TNC-3′, 5′-TNG-3′, and 5′-TNT-3′ around C/T transition type, together with their reverse and complementary motifs around A/G (Fig. 4b and Additional file 1: Fig. S13). Additionally, other four high-frequency motifs were around G/T and A/C transition types, including 5′-ANA-3′ and 5′-TNC-3′ around G/T, together with their reverse and complementary motifs around A/C transition type (Fig. 4b and Additional file 1: Fig. S13). Given the contributions of the four transition types (A/G, C/T, G/T, and A/C transition types) to [AT]-increase mentioned above, the 34 high-frequency motifs could represent major mutation types resulting in [AT]-increase from wild progenitors to bread wheat.

### The fastest [AT]-increase of D subgenome contributes to the highest mutation rates

**Fig. 5:**
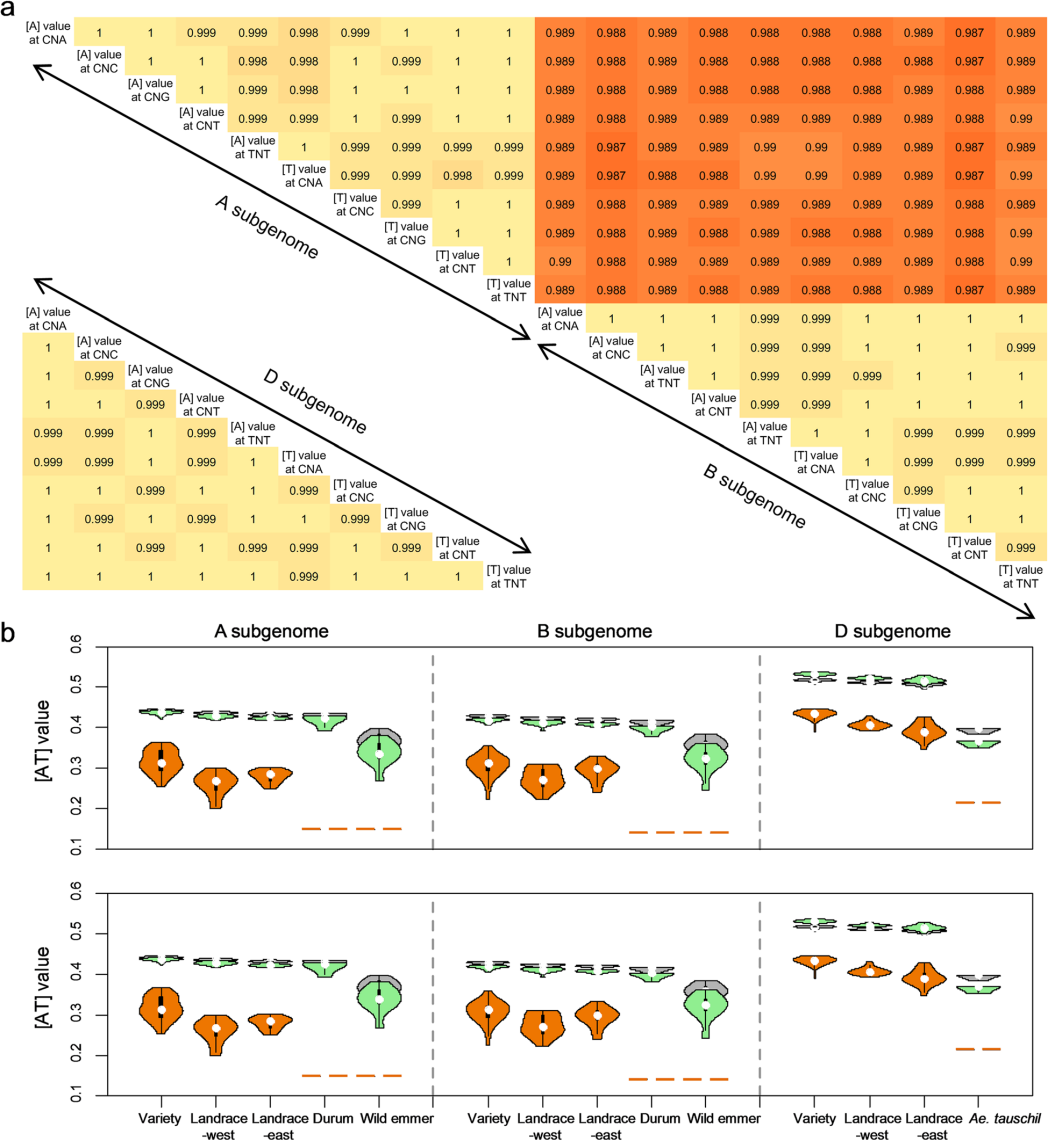
Mutation rates among three subgenomes. **a** Correlation of [A&T] values between any two of these high-frequency motifs around C/T and A/G transition types within each subgenome, and A and B subgenomes. **b** Comparison of mutation rates among three subgenomes using different SNP sets. Gray, green, and yellow violins show [AT] values calculated using all common SNPs, common SNPs at 5 motifs around C/T and A/G transition types, and bread-wheat-private SNPs at 5 motifs around C/T and A/G transition types

Further analyses of base change on the 34 motifs above are conducive to reveal the cause of the fastest [AT]-increase on D subgenome of bread wheat during domestication after allopolyploidization. Here, we focused on 10 motifs with a frequency of more than doubled the expected among the three subgenomes, including 5′-ANG-3′, 5′-CNA-3′, 5′-TNG-3′, 5′-GNG-3′, and 5′-ANA-3′ around C/T type, together with their reverse and complementary motifs around A/G (Fig. 4b and Additional file 1: Fig. S13). First, we found that the [A&T] values correlated almost perfectly (*r* ≈ 1, and *p* value is essentially 0) between any two of the 10 motifs for each subgenome, based on the [A&T] value of each accession of bread wheat, wild emmer, and *Ae. tauschii* (Fig. 5a). Meanwhile, high [A&T] value correlations (mean of *r* ≈ 0.989) were also detected between A and B subgenomes on the 10 motifs, based on the [A&T] value of each accession of bread wheat and wild emmer. These suggested that there were almost unanimous mutation rates which occurred from G or C to A or T among the 10 motifs within each subgenome from wild progenitors to bread wheat.

We further calculated average [T] values of 5 motifs around C/T transition type, and average [A] values of 5 motifs around A/G transition type for each accession, respectively. For 5 motifs around C/T transition type within A and B subgenomes, the average [T] values were highest in the three bread wheat groups, varieties (43.7% and 41.8%), landrace-west (42.8% and 41.2%), and landrace-east (42.3% and 40.8%), followed by durum (41.9% and 39.7%), whereas wild emmer had the lowest average [T] values at 33.6% and 32.0% separately (Fig. 5b and Additional file 1: Fig. S14). Meanwhile, the largest average [T] values were detected in D subgenome of three bread wheat groups, varieties (53.1%), landrace-west (52.2%), and landrace-east (51.6%), and *Ae. tauschii* had lower [AT] value at 32.6% (Fig. 5b and Additional file 1: Fig. S14). By comparison, there was the largest [T]-difference on D subgenome (16.3%) between bread wheat and *Ae. tauschii* than these on A (9.5%) and B (9.6%) subgenomes between bread wheat and wild emmer. Almost identical [AT] values were identified between each of the 5 motifs around A/G types and their corresponding reverse and complementary motifs around C/T (Fig. 5b). The results suggested that high-frequency mutations occurred from G or C to A or T on the 10 motifs during bread wheat domestication after allopolyploidization, which eventually resulted in genome-wide [AT]-increase in bread wheat. Additionally, the highest [AT]-increase of D subgenome could be caused by the most incidences of G to A and C to T transitions on the 10 motifs.

To further verify the mutation rate differences between D subgenome and the other two subgenomes on the 10 high-frequency mutation types mentioned above, we compiled a set of bread-wheat-private variants that occurred as relatively recent mutations during bread wheat domestication after allopolyploidization (see the “Methods” section). A total of 2,278,416, 2,726,435, and 3,132,907 bread-wheat-private SNPs were identified within A, B, and D subgenomes, respectively. A/G and C/T were also two major transition types within bread-wheat-private SNPs, occupying the same frequencies in A (35.1% and 35.1%), B (34.1% and 34.1%), and D subgenomes (34.3% and 34.3%) (Additional file 1: Fig. S15). Another two transition types (A/C and T/G) related to [AT]-increase showed similar frequency (≈ 9.0%) among three subgenomes (Additional file 1: Fig. S15). For 96 motifs around the 6 transition types, the frequency was identical among three subgenomes. Among them, the frequency of 10 motifs around C/T and A/G above was twice that expected as well (Additional file 1: Fig. S16). The results from bread-wheat-private SNPs further confirmed that three subgenomes shared same mutation types, and the 10 motifs at A/G and C/T transition types were the major contributors to [AT]-increase during bread wheat domestication after allopolyploidization.

Next, we assessed the mutation rate differences on the 10 high-frequency motifs among the three subgenomes using these bread-wheat-private SNPs. By comparison with donor allele information, the average frequency of C to T transitions at 5 motifs around C/T was 14.6% and 15.6% in A and B subgenomes separately from wild emmer to bread wheat (Fig. 5b and Additional file 1: Fig. S17). As expected, D subgenome showed the highest frequency of C to T transitions at the 5 motifs around C/T (20.4%) (Fig. 5b and Additional file 1: Fig. S17). Almost identical [AT] values were identified between each of the 5 motifs around A/G types and their corresponding reverse and complementary motifs around C/T. The results further confirmed the mutation rate difference between D subgenome and the other two subgenomes during bread wheat domestication after allopolyploidization.

### The fastest [AT]-increase on D subgenome is determined by a joint repair system across the whole genome

The data from analysis of multiple base-composition value at genome-wide, signal chromosome or a set of subsampling level, demonstrated that [AT] value across polymorphic sites can be regarded as a genome phenotype, which correlate almost perfectly with the first principal component (PC1) values from PC analysis of the SNP data [11, 12]. However, the distinct [AT] values and [AT]-increase across polymorphic sites were identified among A, B, and D subgenomes within bread wheat in this study, which agreed with the known population structure and phylogenetic relationships among three subgenomes [6]. Further PC analyses of each of the three subgenomes indicated that there was a strong correlation between [AT] value and PC1 in A (*r* = − 0.914), B (*r* = − 0.898), and D (*r* = − 0.986) subgenomes, respectively. Hence, it is appropriate that the [AT] values of A, B, and D subgenomes represent the phenotypes of corresponding subgenome.

**Fig. 6:**
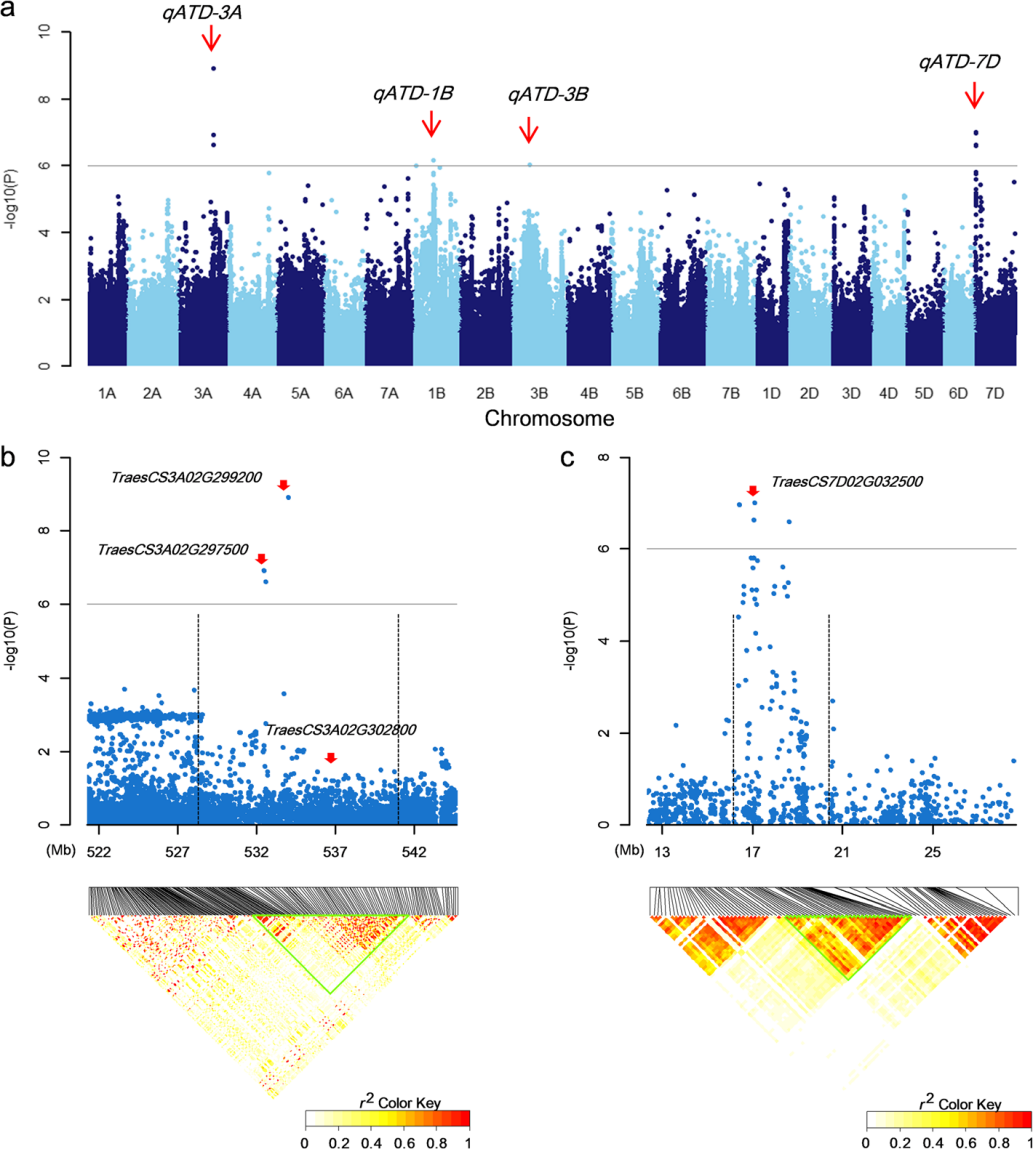
Determination of candidate genes for [AT] value of D subgenome. **a** Manhattan plot in the bread wheat population for [AT] value of D subgenome. Red arrows mark four signals identified in this study. Identification of candidate genes within top two association loci *qATD-3A* (**b**) and *qATD-7D* (**c**). The top of each panel shows the entire association locus identified by GWAS using 63 bread wheat accessions and LMM, and *X*-axis indicates the physical position (Mb) of the region in the bread wheat genome. Negative log10-transformed *p* values are plotted on the vertical axis, in which dots show positions and −log(*p*) values of all SNPs within this association locus. Gray horizontal line shows the threshold at −log(*p*) = 6, and dotted vertical line indicates flanks of association loci. At the bottom of each panel, LD heatmap is plotted to show the possible candidate internal of corresponding association locus. Red arrows show the position of candidate genes

To explore the associated loci underling mutation rate of D subgenome, genome-wide association study (GWAS) was performed by using the [AT] values of D subgenome as its genome phenotype (see the “Methods” section). A total of four associated loci underlying [AT] value of D subgenome (*qATD-3A*, *qATD-1B*, *qATD-3B*, and *qATD-7D*) were detected, containing 10 associated SNPs over the threshold at −log(*p*) = 6 (Fig. 6a and Additional file 1: Fig. S18). One associated signal was on A and D genomes separately, whereas two were on B subgenome. The results suggest that the base composition of D subgenome is not only determined by itself. Linkage disequilibrium (LD) analysis was performed for the top two signals (*qATD-3A* and *qATD-7D*), and the two candidate intervals were defined into 12.5 Mb and 4.2 Mb on chromosomes 3A and 7D, respectively (Fig. 6b, c). There are a total of 94 and 100 annotated genes within *qATD-3A* and *qATD-7D*, respectively. Given that the DNA repair genes are normally expressed in whole organism with no tissue specificity, we further screened 32 and 9 candidates with stable expression (TPM > 1) in roots and shoots of bread wheat (Additional file 2: Table S2). Of all these genes, four (*TraesCS3A02G297500*, *TraesCS3A02G299200*, and *TraesCS3A02G302800* for *qATD-3A* and *TraesCS7D02G032500* for *qATD-7D*) were turned to be highly likely candidates for the QTL based on their functional annotations. *TraesCS3A02G297500* is predicted to encode a protein structurally similar to the multiubiquitin-binding protein RAD23, whose homologous gene HEMERA is involved in the repair of damaged DNA induced by solar ultraviolet [36, 37]. *TraesCS3A02G299200* encodes a nuclear coiled-coil protein, a homolog of which, LINC1-4, is involved in the determination of plant nuclear structure in *Arabidopsis thaliana* [38–40]. Meanwhile, *TraesCS3A02G302800* is predicted to encode a tRNA (guanine-*N*(1)-)-methyltransferase G, and *TraesCS7D02G032500* encodes a DNA topoisomerase-like protein G. Further studies are essential to determine the function of these genes in bread wheat, although we have provided some credible information to support their impact on base composition. Taken together, the results provide several genomic intervals and possible key candidates for further revealing the molecular mechanism underlying [AT]-increase pattern of D subgenome within bread wheat.

## Discussion

### PR2 and [AT]-increase remain on three bread wheat subgenomes during domestication after allopolyploidization

Compared with other crops, the speciation and following global domestication of bread wheat were completed in a very short time [1]. For the region of southeastern Turkey and northern Syria where wild progenitors of bread wheat still are grown today, these accessions provide an opportunity to get insight into the genome change following allopolyploid domestication. Hybrid speciation has played a very important role in plant evolution and speciation [3], and domestication largely involves selection of favorable alleles from standing allelic variation in wild ancestors [41]; however, understanding of how plant genomes have changed in the process remains limited.

PR2 and [AT]-increase seem to be the generic rules of all double-stranded DNA genomes at the levels of genome and polymorphic sites [11, 15], even in bread wheat, a relatively new species. Our studies show that there is not a significant impact on PR2 and [AT]-increase from generation of new mutation, insertions and deletions of chromosomal fragment, genome-wide recombination, and massive alien introgressions during the 6000–10,000 years of bread wheat speciation and domestication [6]. The no-strand-bias mutation hypothesis was proposed to explain PR2, that is, a randomly occurred mutation on one DNA strand must generate a complementary base on the basis of PR1 (A = T and C = G), and the random and paired emergences can ultimately result in PR2 [42, 43]. Meanwhile, two interpretations have been proposed to explain the [AT]-increase [11]. One is that, as a result of lower effective population size, populations after a bottleneck (polyploid speciation or domestication) may have fixed A&T mutations more frequently than the basal groups. The other interpretation is that the DNA repair genes are likely to affect the number of de novo mutations which differed in various lineages and a greater total number of mutations could also cause an increased A&T. Here, our studies provide the comparison of base composition between derived groups (bread wheat) and basal groups (wild progenitors of bread wheat), which demonstrated all these patterns of base-composition change in bread wheat in the process of domestication after allopolyploidization. Further de novo mutation accumulation experiments with a set of genealogical materials would provide evidence to the second interpretation.

### Distinct [AT]-increase between D subgenome and other two subgenomes of bread wheat shows gradual integration of multiple DNA repair systems from its wild progenitors

To date, analysis of data from each of the 8 species groups demonstrated identical [AT]-increase on each chromosome from basal group to derived group [11]. One interesting finding in this study is the fastest [AT]-increase on D subgenome than those on A and B subgenomes from wild progenitors to bread wheat. Actually, the differential [AT]-increase among three bread wheat subgenomes occurs at the same time, which suggests distinct subgenome change pattern within the allopolyploid bread wheat during domestication. Our studies further ruled out the main effect on the differential [AT]-increase among three bread wheat subgenomes from special chromosomal internals, functional genes, sweep regions, and mutation types. Finally, the highest mutation rates of D subgenome are identified, which result in its fast [AT]-increase within bread wheat genome during domestication after allopolyploidization. Although we have confirmed the highest mutation rates of D subgenome by using common SNP set and bread-wheat-private SNP set, the fundamental cause of the fastest [AT]-increase on D subgenome has yet to be thoroughly analyzed. One further question to be asked is: how the newly integrated repair systems regulate the mutation rates of bread wheat genome? First extreme possibility is the mutation rates of D subgenome are determined by itself, as the case for the A and B subgenomes. Another extreme possibility is repair systems from wild progenitors of bread wheat are fully integrated as a new repair system, which regulate the whole genome of bread wheat. The third possibility is that there is no fully integrated repair system to determine the mutation rate of whole genome, and the mutation rate of each subgenome is controlled by itself to some extent. Using [AT] value of D subgenome as the phenotype, genome-wide scans identify a set of putative candidate loci distributed across the whole genome. The results exclude the first two possibilities mentioned above, but a more plausible explanation may require a more thorough understanding of the genetic basis and network of the DNA repair system of bread wheat.

### Distinct [AT]-increase between D subgenome and other two subgenomes of bread wheat could be caused by wide-ranging genome modifications after allopolyploidization

Hybrid speciation is a well-established and fast speciation mode in plants [3, 44], but it is accompanied by a long evolution process of diploidization [45]. Diploidization is thought to occur by genome modifications including chromosomal rearrangement, gene loss, gene conversion, subgenome dominance, and expression divergence between duplicate copies [46–48]. During diploidization, two sets of genomes from different parents gradually integrate in many aspects, such as codon usage, express pattern, and DNA repair system. The hypothesis is reasonable, due to that many studies suggest that most species of flowering plants and vertebrates have descended from ancestors who doubled their genomes, from either autopolyploidy or allopolyploidy [49–52]. And now, they share same rules among their respective chromosomes on codon usage [53, 54], mutation rate, and nucleotide pattern [12]. In other words, diploidization of polyploids requires distinct but convergent changes of subgenomes from different wild progenitors in order to construct a unified whole-genome management system. Large or small differences in expression pattern [31, 55], DNA methylation [56], gene loss [57], nucleotide pattern and mutation rate among three bread wheat subgenomes support that the recently formed hexaploid species bread wheat is undergoing the process of diploidization. Recent research showed that asymmetric breeding selection on key agricultural traits may accelerate the diploidization proceeding of bread wheat genome [58]. Compared with the other speciation modes (such as allopatric speciation, sympatric speciation, peripatric speciation, and parapatric speciation), genome duplications (including autopolyploidy or allopolyploidy) provide the raw material for increased complexity [45, 49]. And wide-ranging genome modifications after polyploidization could be important factors leading to distinct subgenome changes [48, 59], including nucleotide patterns among three subgenomes of bread wheat.

## Conclusions

Our study identified the genome/subgenome-wide base composition of bread wheat and its wild progenitors, and detected distinct nucleotide patterns among three subgenomes of bread wheat during domestication after allopolyploidization. Further researches from multiple perspectives show that the fastest [AT]-increase of D subgenome is caused by its high mutation rate. And the highest mutation rate on D subgenome may be involved in DNA repair systems distributed on three subgenomes of bread wheat.

## Methods

### Genome and sequence information of bread wheat and its wild progenitors

The completed reference genomes of bread wheat, durum, wild emmer, and *Ae. tauschii* were obtained from the corresponding public database [31–34]. The numbers of four base types and the miss base “N” were calculated by in-house Python scripts. We obtained the [AT] value as the ratio of the number of A and T to total number of four base types. To determine the statistical significance on [AT]-difference between wild and domesticated accessions for each subgenome, we performed random shuffling of 100 iterations of [AT] value of 3.6 billion bases for each species and further independent-sample *T* test.

The data for the polymorphic site analysis were obtained from a recently published study, including 84,594,994 SNPs of 93 accessions from worldwide populations of bread wheat, durum, wild emmer, and *Ae. tauschii* [6]. There are clear patterns for geographical distribution and evolutionary relationships among these accessions according to the original publications. After removing SNPs with missing rates > 20% and minor allele frequency (MAF) < 5%, a common SNP set is constructed for all analysis except for bread-wheat-private SNP analyses, including 16,444,250, 16,098,930, and 9,218,675 in A, B, and D subgenomes, respectively. Additionally, following the reported strategy [17], we defined the bread-wheat-private SNP as it is variable only in bread wheat, but not variable in wild emmer, durum, and *Ae. tauschii*. The bread-wheat-private SNP set contains 2,278,416, 2,726,435, and 3,132,907 SNPs in A, B, and D subgenomes, respectively.

### Base composition across polymorphic site

Following the procedure published in a previous study [11], we calculated the base compositions across polymorphic site for each of 93 accessions. For each base, the homozygotes were counted as 1, while the heterozygotes as 0.5. The sum across all SNPs was recorded as the corresponding base value for each accession. The [AT] value was calculated as the fraction of SNP alleles that are either base A or T. To scan the base-composition distribution across whole genome, we calculated the [AT] values of each 2-Mb chromosomal sliding window with the sliding step at 1 Mb. The distribution of recombination is from previous report [31], including 105 differential-recombination zones on 21 bread wheat chromosomes. We calculated and compared the base compositions of 5 zones on each chromosome at the chromosomal window level.

### Base composition among different genomic functional annotation sets

By using software SnpEff v4.3t [35], we classified all SNPs into 7 genomic annotation sets, including synonymous, missense, intronic, UTRs, gene-proximal, intergenic, and other genic SNPs. We then counted the proportion of each of the 7 sets. Given the possible impact on biological function, intergenic and gene-proximal sets were combined into non-genic SNP set and the other five original genomic annotation sets were combined into genic SNP set. Base composition across polymorphic sites was calculated for intergenic SNP set, missense SNP set, genic SNP set, and non-genic SNP set, respectively. For comparison of the [AT] values from different SNP sets, we randomly sampled an equal number of SNPs from intergenic and missense SNP sets, and from non-genic and genic SNP sets, respectively.

The data of selective regions are from recent report [6], including 547 domestication-related selective sweeps (192, 146, and 209 in A, B, and D subgenomes, respectively). For each chromosome, we picked out all SNPs within sweep regions and non-sweep regions, respectively. And then, base composition across polymorphic sites was calculated for SNPs within selective sweep and non-selective sweep regions separately. For chromosome 1B, there was only one selective sweep containing 6 SNPs. Given the possible bias resulting from less SNPs, we did not perform the comparison of [AT] values between the selective sweep and non-selective sweep on chromosome 1B.

### Mutation type and mutation rate related to [AT]-increase

SNPs were divided into 6 transition types, including A/C, A/G, A/T, C/G, C/T, and G/T. We counted the frequency of each transition at all SNPs to determine which was the major mutation type from wild progenitors to bread wheat. For each transition type, the total number of each base type possessed by each accession was counted and divided by the total number of polymorphic sites at corresponding transition type except for the miss base “N.” And further proportional increases in A or T of 4 transition types (A/C, A/G, C/T, and G/T) from wild progenitors to bread wheat were counted to show the mutation rate of corresponding mutation type.

To examine the effect of sequence context of SNPs on mutation type and mutation rate, directly adjacent upstream and downstream bases for each SNP site were extracted from reference genome of bread wheat. There were 96 possible tri-nucleotide motifs around 6 transition types. We counted the frequency of each motif at all SNPs to determine which was the major mutation type from wild progenitors to bread wheat at tri-nucleotide motif level. For each motif, the total number of each motif possessed by each accession was counted and divided by the total number of polymorphic sites at corresponding transition type except for the miss base “N.” For 10 high-frequency motifs around A/G and C/T, the proportional increases in A or T from wild progenitors to bread wheat were counted to show the mutation rate of corresponding motif.

### Association mapping for [AT] value of D subgenome

Because of the non-independence of SNPs caused by strong linkage disequilibrium (LD), it is usually confounding to evaluate population structure [60, 61]. Independent SNP numbers of 63 bread wheat were determined by PLINK (window size 50, step size 50, *r*^2^ ≤ 0.3) [62]. Finally, a total of 1,239,779 unimputed SNPs were extracted for association mapping, with minor allele frequency (MAF) ≥ 5%, missing rate ≤ 20%, and *r*^2^ of LD ≤ 0.3. Genome-wide association study (GWAS) was performed by FaST-Lmm program [63]. Population structure was modeled as a random effect in LMM (linear mixed model) using the kinship (K) matrix, and we found that it was enough to control for spurious associations, because there were no inflated *p* values and the majority (95%) of markers exhibited *p* values equal to or smaller than the expected with accordance null hypothesis. For an appropriate threshold, independent SNP numbers were calculated, given that it might be too strict for significant association detection when the threshold was derived from the total number of markers [60, 61]. Finally, the threshold to control the type I error rate was defined at −log(*p*) = 6 after Bonferroni-adjusted correction [64].

### Screening of candidate genes for [AT] value of D subgenome

In order to identify candidate genes in the associated loci, LD heatmaps surrounding peaks in the GWAS were constructed using the R package “LD heatmap” [65]. By using pairwise LD correlations (*r*^2^ > 0.6), we estimated candidate regions of two peak association signals [66]. To narrow down the candidate number within the associated loci, we further investigated the expression of these candidates in root and shoot based on published transcription data [31].

## Supplementary Information

**Additional file 1:** **Fig. S1.** DNA base composition of each chromosome captured by genome sequence of bread wheat (AABBDD, Chinese Spring, blue), durum (AABB, Svevo, red), wild emmer (AABB, Zavitan, green) and *Ae. tauschii* (DD, AL8/78, brown). **Fig. S2.** [AT] values captured by reported genome sequence of bread wheat, durum, wild emmer and *Ae. tauschii*. **Fig. S3.** [AT] values of randomly sampled bases. **Fig. S4.** Individual-strand DNA base composition parity within each of three bread wheat subgenomes. **Fig. S5.** Individual-strand DNA base composition parity within each chromosome of bread wheat. **Fig. S6.** Base composition distribution among different chromosome regions of bread wheat and its wild progenitors. **Fig. S7.** The distribution of [AT]-difference between bread wheat and its wild progenitors along the bread wheat genome. **Fig. S8.** [AT]-differences between bread wheat and its wild progenitors among differential-recombination zones. **Fig. S9.** [AT] values of randomly sampled intergenic and missense SNPs (left), and non-genic and genic SNPs (right). **Fig. S10.** [AT] values captured by SNPs from selective sweeps (left) and non-selective sweeps (right) among three subgenomes. **Fig. S11.** Frequency of 6 SNP transition types on common SNP set of A **(a)**, B **(b)**, and D **(c)** subgenome. **Fig. S12.** Base values at each transition type of A/G (top), A/C (middle), and C/G transition type (bottom) within bread wheat and its wild progenitors. **Fig. S13.** Frequency of 48 motifs at A/G (top), A/C (middle) and G/C transition type (bottom). **Fig. S14.** [A&T] values at 10 motifs around C/T and A/G transition types. **Fig. S15.** Frequency of 6 SNP transition types using bread wheat-private SNP set on A **(a)**, B **(b)**, and D **(c)** subgenome. **Fig. S16.** Frequency of 96 motifs at 6 transition type using bread-wheat-private SNPs. **Fig. S17.** [A&T] values at 10 motifs around C/T and A/G transition types of bread-wheat-private SNPs. **Fig. S18.** Quantile-quantile (Q-Q) plot for [AT] value of D subgenome.

**Additional file 2:** **Table S1.** The comparison of [AT] values across ploymorphic sites among bread wheat and its wild progenitors. **Table S2.** Expression of candidate genes within *qATD-3A* and *qATD-7D.*

## Data Availability

The data for the polymorphic site analysis are obtained from published studies [6, 31, 67, 68]. The NCBI accessions are PRJNA476679, PRJNA329335 (SRR5170323, SRR5184282, and SRR5184283), and PRJNA392179 (SRR5815659, SRR5817288, SRR5817289, and SRR5817290), respectively. RNA-seq data are also from published study [31], which are at NCBI under accession code SRP028357.
