# Supplementary material for: Distinct nucleotide patterns among three subgenomes of bread wheat and their potential origins during domestication after allopolyploidization
Source: BMC Biol. 2020 Dec 2;18:188. doi: 10.1186/s12915-020-00917-x (PMC7713161; doi:10.1186/s12915-020-00917-x)
Supplement: Supplementary file 1 — Additional file 1: Fig. S1. DNA base composition of each chromosome captured by genome sequence of bread wheat (AABBDD, Chinese Spring, blue), durum (AABB, Svevo, red), wild emmer (AABB, Zavitan, green) and Ae. tauschii (DD, AL8/78, brown). Fig. S2. [AT] values captured by reported genome sequence of bread wheat, durum, wild emmer and Ae. tauschii. Fig. S3. [AT] values of randomly sampled bases. Fig. S4. Individual-strand DNA base composition parity within each of three bread wheat subgenomes. Fig. S5. Individual-strand DNA base composition parity within each chromosome of bread wheat. Fig. S6. Base composition distribution among different chromosome regions of bread wheat and its wild progenitors. Fig. S7. The distribution of [AT]-difference between bread wheat and its wild progenitors along the bread wheat genome. Fig. S8. [AT]-differences between bread wheat and its wild progenitors among differential-recombination zones. Fig. S9. [AT] values of randomly sampled intergenic and missense SNPs (left), and non-genic and genic SNPs (right). Fig. S10. [AT] values captured by SNPs from selective sweeps (left) and non-selective sweeps (right) among three subgenomes. Fig. S11. Frequency of 6 SNP transition types on common SNP set of A (a), B (b), and D (c) subgenome. Fig. S12. Base values at each transition type of A/G (top), A/C (middle), and C/G transition type (bottom) within bread wheat and its wild progenitors. Fig. S13. Frequency of 48 motifs at A/G (top), A/C (middle) and G/C transition type (bottom). Fig. S14. [A&T] values at 10 motifs around C/T and A/G transition types. Fig. S15. Frequency of 6 SNP transition types using bread wheat-private SNP set on A (a), B (b), and D (c) subgenome. Fig. S16. Frequency of 96 motifs at 6 transition type using bread-wheat-private SNPs. Fig. S17. [A&T] values at 10 motifs around C/T and A/G transition types of bread-wheat-private SNPs. Fig. S18. Quantile-quantile (Q-Q) plot for [AT] value of D subgenome. [file 12915_2020_917_MOESM1_ESM.docx]

**Fig. S1** DNA base composition of each chromosome captured by genome sequence of bread wheat (AABBDD, Chinese Spring, blue), durum (AABB, Svevo, red), wild emmer (AABB, Zavitan, green) and *Ae. tauschii* (DD, AL8/78, brown).

**Fig. S2** [AT] values captured by reported genome sequence of bread wheat, durum, wild emmer and *Ae. tauschii*.

**Fig. S3** [AT] values of randomly sampled bases. For each subgenome, the [AT] values are calculated with 3.6 billion bases of wild emmer, *Ae. tauschii*, durum and bread wheat, respectively. The equal number of SNPs are randomly sampled from the corresponding genome sequence data. The average [AT] values over 100 iterations are plotted.

**Fig. S4** Individual-stand DNA base composition parity within each of three bread wheat subgenomes. All 93 accessions are denoted by dots with different colors (red: wild emmer, pink: durum, skyblue: bread wheat landrace and dark green: bread wheat variety). [A] and [C] values of each accession are plotted to show PR2 across polymorphic sites ([A] + [C] ≈ 0.5 is due to [A] ≈ [T], [G] ≈ [C], and [A] + [T] + [G] + [C] = 1).

**Fig. S5** Individual-stand DNA base composition parity within each chromosome of bread wheat. All 93 accessions are denoted by dots with different colors (red: wild emmer, pink: durum, skyblue: bread wheat landrace and dark green: bread wheat variety). [A] and [C] values of each accession are plotted to show PR2 across polymorphic sites ([A] + [C] ≈ 0.5 is due to [A] ≈ [T], [G] ≈ [C], and [A] + [T] + [G] + [C] = 1). [AT] increase pattern is exhibited by the separation of dots with different colors.

**Fig. S6** Base composition distribution among different chromosome regions of bread wheat and its wild progenitors. For each 2 Mb sliding window, the mean [AT] values of different populations are plotted on each chromosome. The sliding step is 1 Mb. Three groups of bread wheat including variety, landrace-west and landrace-east are colored dark green, green and light green, respectively. Wild emmer and *Ae. tauschii* are colored yellow, when durum is colored red. The five main differential-recombination zones on each chromosome are separated by gray lines in each plot: R1 and R3 (distal regions), R2a and R2b (middle regions); C (proximal region).

**Fig. S7** The distribution of [AT]-difference between bread wheat and its wild progenitors along the bread wheat genome. For each 2 Mb sliding window, the mean [AT]-differences are plotted on each chromosome. The sliding step is 1 Mb. The five main differential-recombination zones on each chromosome are separated by gray lines in each plot: R1 and R3 (distal regions), R2a and R2b (middle regions); C (proximal region).

**Fig. S8** [AT]-differences between bread wheat and its wild progenitors among differential-recombination zones. The letters above the boxes indicate significant difference (*p* < 0.05) when analyzed by Duncan’s test.

**Fig. S9** [AT] values of randomly sampled intergenic and missense SNPs (left), and non-genic and genic SNPs (right). For A and B subgneomes, the [AT] values are calculated with the equal number of SNPs (33,000) from intergenic and missense SNPs, and the euqual number of SNPs (190,000) from non-genic and genic SNPs, respectively. For D subgenome, the [AT] values are from 19,000 intergenic SNPs and 19,000 missense SNPs, and 100,000 non-genic SNPs and 100,000 genic SNPs. These SNPs are randomly sampled from the corresponding SNP sets, and the average [AT] values over 100 iterations are plotted.

**Fig. S10** [AT] values captured by SNPs from selective sweeps (left) and non-selective sweeps (right) among three subgenomes. The letters above the boxes indicate significant differences (*p* < 0.05) when analyzed by Duncan’s test.

**Fig. S11** Frequency of 6 SNP transition types on common SNP set of A **(a)**, B **(b)**, and D **(c)** subgenome. The subgenome-wide SNPs were classified into 6 transition types and frequency for each of the 6 transition types was calculated.

**Fig. S12** Base values at each transition type of A/G (top), A/C (middle), and C/G transition type (bottom) within bread wheat and its wild progenitors. For each transition type, the violins of three subgenomes are plotted together and separated by dashed lines. For each plot, the length of block arrow presents the [A]-difference on corresponding transition type of A or B subgenome between bread wheat and wild emmer, whereas the length of red arrow presents the [A]-difference on corresponding transition type of D subgenome between bread wheat and *Ae. tauschii*.

**Fig. S13** Frequency of 48 motifs at A/G (top), A/C (middle) and G/C transition type (bottom). For each plot, blue, red, and green bars show A, B, and D subgenome, respectively. The numbers 1-16 on Y axis show 16 motifs, 5’-ANA-3’, 5’-ANC-3’, 5’-ANG-3’, 5’-ANT-3’, 5’-CNA-3’, 5’-CNC-3’, 5’-CNG-3’, 5’-CNT-3’, 5’-GNA-3’, 5’-GNC-3’, 5’-GNG-3’, 5’-GNT-3’, 5’-TNA-3’, 5’-TNC-3’, 5’-TNG-3’ and 5’-TNT-3’ in order, where N is corresponding transition type. The horizontal line in each plot indicated the expected frequency at 0.01 (≈ 1/96). The 17 motifs with frequency above the threshold are marked by arrows, including 5 motifs with frequency more than twice the threshold marked by red arrows.

**Fig. S14** [A&T] values at 10 motifs around C/T and A/G transition types. [T] and [C] values at 5 motifs around C/T were plotted on the right, when [A] and [G] values at 5 motifs around A/G were plotted on the left. Different subgenomes were separated by dotted lines in each plot. Bread wheat and its wild progenitors were divided into 6 groups, including bread wheat variety (V), landrace-west (L-W), landrace-east (L-E), durum (D), wild emmer (W) and *Ae. tauschii* (A).

**Fig. S15** Frequency of 6 SNP transition types using bread wheat-private SNP set on A **(a)**, B **(b)**, and D **(c)** subgenome. The subgenome-wide SNPs were classified into 6 transition types and frequency for each of the 6 transition types was calculated.

**Fig. S16** Frequency of 96 motifs at 6 transition type using bread-wheat-private SNPs. For each plot, blue, red, and green bars show A, B, and D subgenome, respectively. The numbers 1-16 on Y axis show 16 motifs, ANA, ANC, ANG, ANT, CNA, CNC, CNG, CNT, GNA, GNC, GNG, GNT, TNA, TNC, TNG and TNT in order, where N is corresponding transition type. The horizontal line in each plot indicated the expected frequency at 0.01 (≈ 1/96). The 34 motifs with frequency above the threshold are marked by arrows, including 10 motifs with frequency more than twice the threshold marked by red arrows.

**Fig. S17** [A&T] values at 10 motifs around C/T and A/G transition types of bread-wheat-private SNPs. [T] and [C] values at 5 motifs around C/T were plotted on the right, when [A] and [G] values at 5 motifs around A/G were plotted on the left. Different subgenomes were separated by dotted lines in each plot. Bread wheat and its wild progenitors were divided into 6 groups, including bread wheat variety (V), landrace-west (L-W), landrace-east (L-E), durum (D), wild emmer (W) and *Ae. tauschii* (A).

**Fig. S18** Quantile-quantile (Q-Q) plot for [AT] value of D subgenome. Dotted line show threshold at -log(*P*) = 6.
